# The Effect of a Remote Network Technology Supervised Exercise Program Combined With Drug Treatment for Fibromyalgia: Randomized, Single-Blind, Controlled Trial

**DOI:** 10.2196/71624

**Published:** 2025-06-26

**Authors:** Cuomaoji Zhang, Peijun Zhang, Yuanmeng Zhao, Yuntao Liu, Yun Hu, Zihan Zhu, Hong Xiao

**Affiliations:** 1 Department of Pain Management, West China Hospital of Sichuan University, 37 Guoxue Alley, Wuhou District, Chengdu, 610041, China, 86 18980601980

**Keywords:** fibromyalgia, exercise, remote supervision, pregabalin, duloxetine, efficacy

## Abstract

**Background:**

Fibromyalgia (FM) is a chronic musculoskeletal pain disorder that is seldom reported in China. Recent studies have focused on nondrug treatments, particularly physical therapy, as an alternative to treatments using medication. With the rise of smartphones and mobile communication, mobile health technology has become a significant area of study.

**Objective:**

This study aims to explore whether using remote network applications to supervise patient exercise, in combination with medication, can improve FM pain. It builds on previous research that focuses on drug treatments and offers insights into individualized exercise therapy for FM.

**Methods:**

The study used a prospective, randomized controlled design with 80 participants, who were divided into 2 groups: supervised and unsupervised. Both groups received a drug regimen: oral pregabalin (75‐150 mg twice daily) and duloxetine (30‐60 mg once daily). The supervised group followed exercise routines with guidance from web-based rehabilitation therapist via a remote network application, while the unsupervised group exercised without supervision. The study was blinded to the participants. Primary outcomes were pain levels over the past 24 hours as measured by the Brief Pain Inventory (BPI). Secondary outcomes included pain relief, sleep improvement, quality of life, and adverse event occurrences. Observations were made at the start of treatment (T0), 1 month after treatment (T1), and 3 months after treatment (T3).

**Results:**

We recruited 80 participants, evenly divided into 2 groups, from August 2022 to December 2023 at West China Hospital of Sichuan University. Comparisons of the 2 groups were performed using analysis of variance and Bonferroni post hoc analyses (SPSS version 25 for Windows, *P*<.05 considered as significant). Compared with T0, the Widespread Pain Index (WPI), symptom severity score (SSS), and BPI (pain on average, least pain in past 24 h, pain right now) scores of the 2 groups of patients with fibromyalgia at T1 were significantly lower. Compared with T0, the WPI, SSS, BPI (pain on average, worst pain in past 24 h, least pain in past 24 h, pain right now), and Fibromyalgia Impact Questionnaire scores of the 2 groups of patients at T3 were significantly lower. The WPI, SSS, BPI (pain on average, worst pain in past 24 h, pain right now), and Pittsburgh Sleep Quality Index scores of the 2 groups at T3 were significantly lower than at T1. However, the significance of some of the data did not exist after Bonferroni correction. The changes in scores from T0 to T1 (T1–T0), from T0 to T3 (T3–T0), and from T1 to T3 (T3–T1) in the supervised group were all less statistically significant compared to the unsupervised group.

**Conclusions:**

The study showed that exercise combined with drug therapy can significantly improve the prognosis of FM, including pain relief, better sleep, and better overall quality of life; long-term supervised exercise training is more effective in improving FM symptoms and is safer and more reliable than unsupervised exercise.

## Introduction

Fibromyalgia (FM) is a chronic musculoskeletal pain syndrome whose pathogenesis and pathophysiological processes have not yet been fully elucidated. It is characterized by widespread chronic musculoskeletal pain, hyperalgesia in specific areas, and a variety of physical and psychological disorders, such as fatigue, sleep disorders, anxiety, depression, cognitive dysfunction, headache, and gastrointestinal dysfunction [1]. It has been reported that the global prevalence of FM is 2.1%. The incidence of FM in women is higher than in men, with a ratio of 4:1. There is still a lack of epidemiological data on the disease in China [2]. Many patients with fibromyalgia are diagnosed late. It often takes about 2 years from the first onset of symptoms to the accurate diagnosis. At the same time, most patients have symptoms such as anxiety and depression, which significantly affect their quality of life [3].

Among the existing treatments, the anticonvulsant drug pregabalin and the antidepressant duloxetine are the 2 that have been widely used to treat FM in recent years. They can relieve pain through different pharmacological effects. Therefore, the combination of them produces additive effects [4]. A previous study also demonstrated the efficacy and safety of duloxetine combined with pregabalin in the treatment of FM [5]. Relevant studies found that some patients with fibromyalgia who have been taking medication for a long time have adverse reactions such as dizziness, drowsiness, nausea, and vomiting, and some even stop taking the drug on their own because they cannot tolerate the adverse reactions [6,7], which results in the continued use of medication. It is unable to meet the patient’s psychological expectations.

In recent years, there has been an increase in research on nondrug treatments, like physical therapy, as an alternative treatment for FM. Studies have shown that exercise therapy can improve symptoms such as pain, sleep disorders, anxiety, and depression in patients with fibromyalgia and has the dual advantages of being economical and safe [8,9]. Many FM management guidelines recommend that patients with fibromyalgia start exercise therapy as soon as possible after a clear diagnosis and report that aerobic exercise and resistance training effectively improve pain and physical function [9]. Additionally, group physical exercise has gained attention for its potential to enhance patient compliance and outcomes. Participating in group activities fosters social support, motivation, and accountability, which are critical for maintaining long-term adherence to exercise programs. Several studies have demonstrated that group-based interventions improve adherence rates and overall treatment efficacy compared to individual exercise regimens [10]. It is reported that nearly 40% of patients with fibromyalgia are unwilling to exercise because of pain. This psychological fear of exercise leads to physical weakness because less exercise leads to greater physical weakness, which in turn leads to more pain and depression, forming a vicious cycle [10,11]. Therefore, integrating group physical exercise into FM management strategies may offer a promising approach to addressing both the physical and psychological challenges faced by patients. However, the window of exercise therapy for FM seems to be very narrow. Too little exercise will not produce benefits, while too much will aggravate symptoms [12]. In addition, patients often have poor compliance. Most patients with fibromyalgia may experience temporary pain, fatigue, and other symptoms when they first start exercising, as well as chronic musculoskeletal injuries [13], which may lead to the inability to adhere to exercise therapy.

Since patients with fibromyalgia cannot perform active movements, designing a remotely supervised treatment strategy is necessary. Remote network supervision technology is one of the feasible methods that could be used [14,15]. With the widespread popularity of smartphones and the rapid development of mobile communication technology, mobile medical technology based on applications has become a hot research area. This remote information-processing program uses telephone, video, and computer technology to replace the traditional face-to-face diagnosis and treatment between doctors and patients [16]. Remote network supervision technology is a telemedicine application focusing on chronic pain and orthopedic functional exercise rehabilitation. It provides comprehensive exercise rehabilitation for orthopedic, cervical, spondylotic, lumbar and leg pain, and spinal-related diseases. The application has the following functional features:

1. Intelligence: Based on intelligent data collection, follow-up, and machine learning depth (artificial intelligence), it implements the management path of chronic pain rehabilitation.
2. Personalization and refinement: Customize personalized and refined exercise and follow-up plans according to patient needs.
3. Real-time home management: Remotely monitor patient recovery, push medication reminders, complete response collection, and achieve home management.
4. Data collection and analysis: Patients provide real-time data feedback through artificial intelligence, applications, and sensor systems, and the background integrates and analyzes abnormal situations. There is no relevant research on whether the above functions can be used for FM management, whether they can improve the ability of patients with fibromyalgia to perform active movements, and whether the combined use of drug therapy can improve the overall treatment efficacy of FM.

Given this, the objectives of our project are using a remote network application to efficiently supervise and guide patients’ exercise in combination with drug treatment (pregabalin and duloxetine) and observing whether it can further improve the clinical symptoms of patients with fibromyalgia, such as pain and sleep disorders, and improve their quality of life, providing new ideas for the individualized treatment of FM.

## Methods

### Ethical Considerations

This study is a single-center, single-blind, randomized controlled trial, which has been approved by the Biomedical Ethics Committee of West China Hospital of Sichuan University (ethics approval number: 2022 Review [650]). The trial was registered on the Chinese Clinical Trial Registry (ChiCTR2500096370) and was conducted following the ethical principles of the Declaration of Helsinki. Before inclusion in the study, all participants received detailed informed consent and voluntarily signed the informed consent form to ensure that they fully understood and agreed to participate in this study. The study ensured the absolute confidentiality of patient information and privacy, which was also communicated during the signing of the informed consent form. There have been no essential changes to the methods since the study started.

### Participants

We recruited 80 patients aged 20-70 years with a clear diagnosis of FM from August 2022 to December 2023 at West China Hospital of Sichuan University. The diagnostic criteria met the 2016 revision of the American College of Rheumatology 2010/2011 FM diagnostic criteria [17]. In addition, patients’ visual analog scale score was not less than 4. However, those patients who had severe cardiopulmonary disease, severe liver or kidney dysfunction, previous mental illness, opioid use, allergies to trial-related drugs, contraindications to exercise training, or an inability to provide informed consent were excluded.

### Interventions

#### Overview

All of the participants were randomly divided into 2 groups. One was supervised, and the other was unsupervised. Both groups received an initial drug treatment regimen of oral pregabalin (75 mg) twice per day combined with duloxetine (30 mg) once per day. If the pain was not relieved, the dose of pregabalin and duloxetine would be escalated to 150 mg and 60 mg, respectively, as tolerated by the patients. Meanwhile, all participants used a remote network application named Healbone (developed by Jiakang Zhongzhi Technology Company) for individual exercise training. The supervised group finished their training under the web-based supervision of a specialized rehabilitation therapist, while the unsupervised group did not.

**Figure 1.**
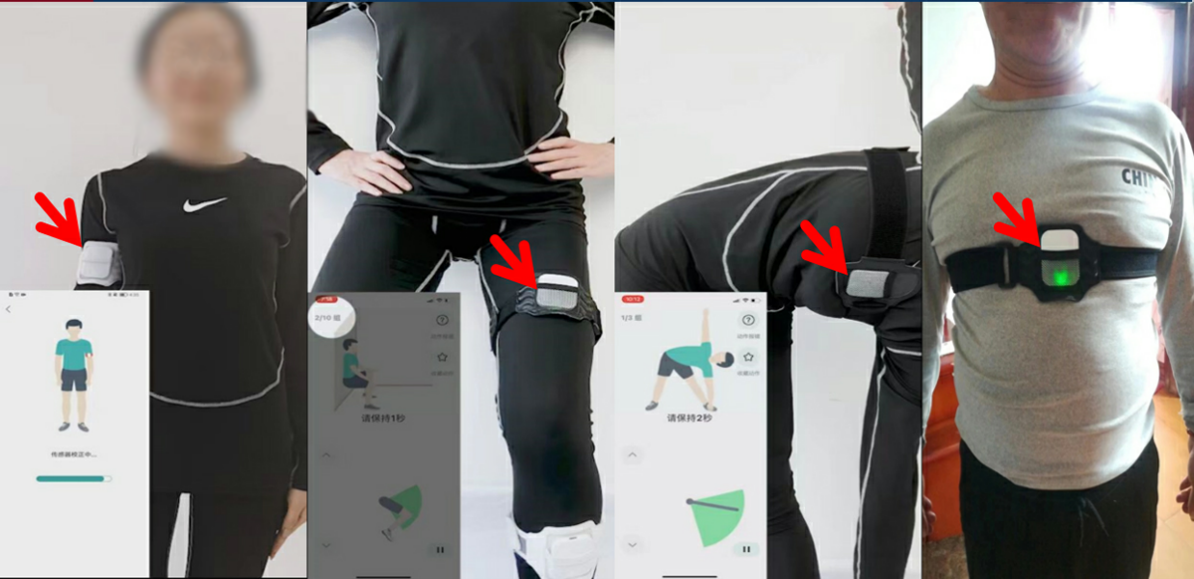
Telemedicine mobile app. The red arrow points to the sensor device. Only by being fixed to the moving body parts could the sensor device upload the sports data to the application terminal when the participant was exercising.

Healbone designed exercises for both groups according to practice guidelines for exercise interventions for patients with fibromyalgia (2021 [18]), combining aerobic, strength, and flexibility exercises. The exercise intensity was gradually increased according to the patient’s tolerance level, ensuring no worsening of symptoms [12]. Patients were instructed to wear a paired sensor device during exercise. The sensor device needed to be fixed to the moving body parts in order to upload the sports data to the application terminal when the participant was exercising (Figure 1).

The details of the exercise regimen are as follows.

#### Aerobic Exercises

Patients performed low-impact aerobic activities such as walking, cycling, or swimming. Each session lasted 0‐30 minutes, with a target heart rate of 60%‐70% of the individual’s maximum heart rate. Aerobic exercises were conducted 3 times per week.

#### Strength Training

Strength exercises focused on major muscle groups using light resistance (eg, resistance bands or bodyweight exercises). Each session included 8‐10 exercises, with 2‐3 sets of 10‐15 repetitions per exercise. Strength training was performed 2 times per week.

#### Flexibility Exercises

Stretching exercises targeted major muscle groups and were performed for 10‐15 minutes per session. Flexibility training was integrated into every exercise session to improve range of motion and reduce stiffness.

#### Progression and Intensity

The intensity of the exercises was gradually increased based on the patient’s tolerance level, ensuring no worsening of symptoms. Progression was monitored weekly, and adjustments were made to maintain a balance between challenge and safety.

#### Session Details

The intervention lasted 12 weeks, with a total of 36 sessions (3 sessions per week). Each session lasted 45-60 minutes, including warm-up, main exercises, and cooldown. Sessions were conducted 3 times per week, with at least 1 rest day between sessions to allow for recovery.

#### Device Description

Patients were required to wear a paired sensor device while exercising. The device, developed by Healbone, was a lightweight, wearable motion sensor that could be fixed to moving body parts (eg, wrist, ankle, or waist). The sensor tracked real-time movement data, including step count, range of motion, and exercise duration. The collected data were automatically uploaded to the application terminal via Bluetooth, allowing for real-time monitoring and feedback (Figure 1). The device was designed to be noninvasive and easy to use, ensuring minimal disruption to the exercise routine.

#### Monitoring and Feedback

The application terminal provided real-time feedback on exercise performance, including adherence to the prescribed intensity and duration. Patients and researchers could access the data to monitor progress and make necessary adjustments to the exercise program.

Both groups of patients downloaded Healbone and received their exercise plan. Both groups received reminders from Healbone to take their medication and a consultation with an online therapist. If the system detected that the daily exercise was not up to the prescribed standard, the supervised group would receive a message from Healbone, and the unsupervised group would not.

### Outcomes

The primary outcome measure was the pain on average in the past 24 hours as determined through Brief Pain Inventory (BPI) scores. The secondary outcomes included the following:

1. Overall pain relief: the highest and lowest real-time scores on the BPI scale, the patient’s Widespread Pain Index (WPI) score, and symptom severity score (SSS)
2. Improvement in sleep, assessed via the Pittsburgh Sleep Quality Index (PSQI) score
3. Improvement in quality of life, assessed via the Fibromyalgia Impact Questionnaire (FIQ) score
4. Incidence of adverse events.

The observation time points were at the beginning of treatment (T0), 1 month after treatment (T1), and 3 months after treatment (T3).

### Sample Size

We used PASS (version 15; NCSS LLC) to calculate the sample size. Our preliminary experiment showed that the pain relief rates of the supervised and unsupervised groups were 80% and 50%, respectively. A total sample size of 40 participants per group was required to reach 90% power with an α of 0.05.

### Randomization

All participants were distributed according to the principle of simple randomization. Excel (Microsoft Corp) generated a random number table. It was taken in a nontransparent bag and was kept by a third party (neither the implementer of the study nor the statistical analyst generated the random allocation sequence and assigned participants to each group). The random number table was not uncovered until all outcomes were observed.

### Blinding

As supervised or unsupervised objects, patients themselves cannot be blinded. However, researchers will not explicitly tell patients about the grouping and how other patients are supervised. After the study implementer enrolled the patients, a dedicated person was assigned to maintain the random number table and group the patients, and follow-up data analysis was completed by a separate data analyst. Blinding was not done until all follow-up was completed and the analysis was ready. For data acquisition, we evaluated the frequency of patient movement through objective data returned by motion sensors and assess the quality of exercise therapy through questionnaire surveys of research participants.

### Statistical Methods

This study was a single-blind, randomized, controlled study. SPSS 25.0 software (IBM Corp) was used to perform statistical analysis on the clinical data of the 2 groups of patients with fibromyalgia. Quantitative data with normal distribution of T0, T1, and T3 were described as mean (SD), while nonnormally distributed quantitative data were described by median and interquartile range for both groups of patients (within-group data analysis). All post hoc multiple comparisons between the 3 time points were adjusted using Bonferroni correction. Level of significance was set at 5%. One-way ANOVA was used to compare the differences and variations between the groups (between-group data analysis). Two-sided tests were used, and *P*<.05 indicated that the differences were statistically significant.

## Results

### Participant Recruitment and Baseline Data

**Figure 2.**
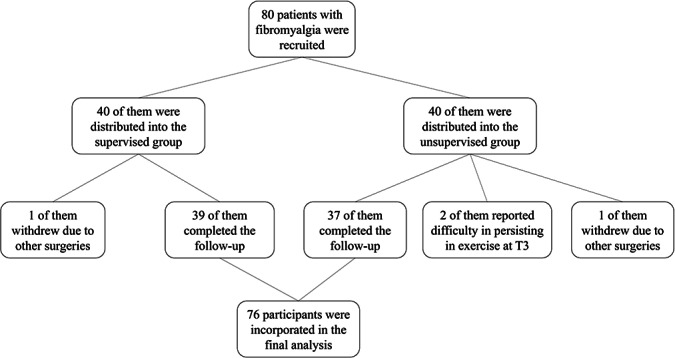
Flow diagram of participant recruitment. T3: 3 months after treatment.

Eighty patients with fibromyalgia who visited the outpatient department of the Pain Department of West China Hospital, Sichuan University, were recruited to this study from August 2022 to December 2023. Data follow-up was terminated when a participant experienced a serious adverse event (such as injury or death), was readmitted to the hospital, or had difficulty maintaining exercise voluntarily. Among them, 2 patients withdrew from the trial due to other surgeries, and 2 found it challenging to persist in exercise at T3. Finally, 76 participants were included, 39 of which were recruited in the supervision group and 37 in the unsupervised group. All patients completed follow-up and were included in the statistical analysis (Figure 2).

We collected participants’ baseline data, including age, gender, weight, and duration of FM. The two groups had no statistical difference in the baseline data of patients with fibromyalgia. Analysis was completed according to the original assigned groups (Table 1).

**Table 1.** Comparison of baseline data between the 2 groups.

Demographic characteristics	Supervision group	Unsupervised group	*P* value
Age (years), mean (SD)	41.38 (11.22)	41.90 (10.79)	.85
Gender (male:female)	14:26	15:25	.82
Weight (kg), mean (SD)	57.69 (9.13)	60.23 (9.22)	.17
Disease duration (years), mean (SD)	3 (1)	3 (2)	.48

### Comparison of Outcomes at T0, T1, and T3 Between the 2 Groups

**Figure 3.**
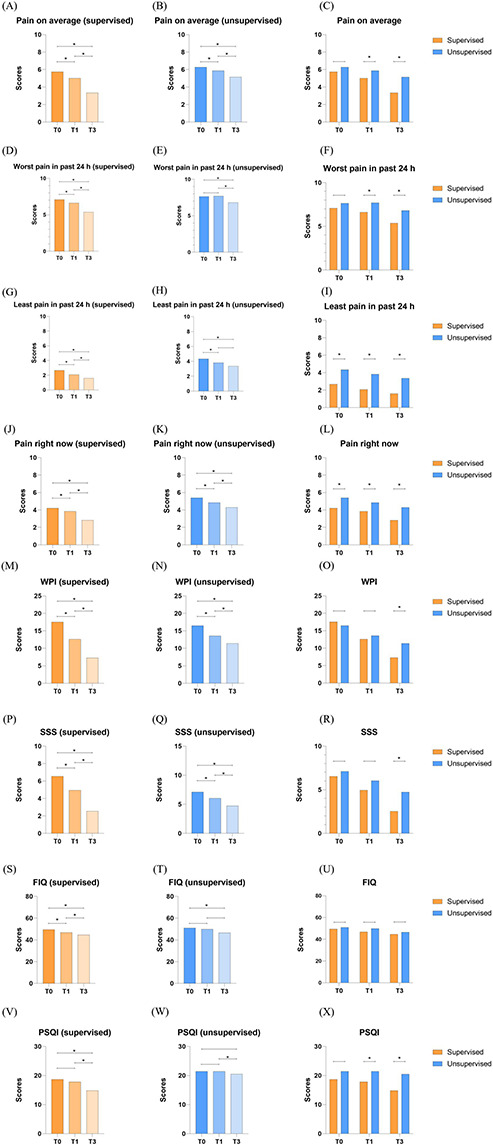
Bar graphs of BPI (pain on average, worst pain in past 24 h, least pain in past 24 h, pain right now), WPI, SSS, FIQ, and PSQI scores. BPI: Brief Pain Inventory; FIQ: Fibromyalgia Impact Questionnaire; PSQI: Pittsburgh Sleep Quality Index; SSS: symptom severity score; WPI: Widespread Pain Index. *P<.05. The specific *P* values are listed in Table 3.

Compared with T0, the WPI, SSS, and BPI (pain on average, least pain in past 24 h, pain right now) scores of the 2 groups of patients with fibromyalgia at T1 were significantly lower. Compared with T0, the WPI, SSS, BPI (pain on average, worst pain in past 24 h, least pain in past 24 h, pain right now), and FIQ scores of the 2 groups of patients with fibromyalgia at T3 were significantly lower. The WPI, SSS, BPI (pain on average, worst pain in past 24 h, pain right now), and PSQI scores of the 2 groups at T3 were significantly lower than at T1. There was no significant difference in outcome measures at the time points not mentioned above. The average pain scores at T1 and T3 were lower in the supervised group than in the unsupervised group. The worst pain scores in the past 24 hours at T1 and T3 were lower in the supervised group than in the unsupervised group. The scores for least pain in the past 24 hours and pain right now at T0, T1, and T3 were lower in the supervised group than in the unsupervised group. The WPI score at T3 was lower in the supervised group than in the unsupervised group. The SSS scores at T1 and T3 were lower in the supervised group than in the unsupervised group. The PSQI score at T1 and T3 was lower in the supervised group than in the unsupervised group. However, after Bonferroni correction, the significant differences in some of the data between the 2 groups of patients at different time points were no longer statistically significant (*P*≥.05; Tables2  3, Figure 3).

**Table 2.** Between-group differences of the supervised versus unsupervised groups across time points (T0, T1, T3); *t* tests with Bonferroni correction^a^.

Evaluation metrics	Supervision group												Unsupervised group											
	T0	T1	*t* value (*df*)	*P* value (adjusted^b^)	T0	T3	*t* value (*df*)	*P* value (adjusted)	T1	T3	*t* value (*df*)	*P* value (adjusted)	T0	T1	*t* value (*df*)	*P* value (adjusted)	T0	T3	*t* value (*df*)	*P* value (adjusted)	T1	T3	*t* value (*df*)	*P* value (adjusted)
Pain on average, mean (SD)	5.74 (2.51)	5.00 (2.28)	3.85 (38)	<.001 (.42)	5.74 (2.51)	3.33 (1.75)	10.54 (38)	<.001 (<.001)	5.00 (2.28)	3.33 (1.75)	9.60 (38)	<.001 (<.001)	6.28 (2.12)	5.87 (1.99)	1.95 (38)	.06 (>.99)	6.28 (2.12)	5.14 (1.51)	4.80 (36)	<.001 (.03)	5.87 (1.99)	5.14 (1.51)	2.98 (36)	.005 (.30)
Worst pain in past 24 h, mean (SD)	7.08 (2.73)	6.62 (2.74)	2.43 (38)	.02 (>.99)	7.08 (2.73)	5.36 (2.05)	5.72 (38)	<.001 (.01)	6.62 (2.74)	5.36 (2.05)	4.85 (38)	<.001 (.09)	7.64 (2.53)	7.69 (2.40)	−0.28 (38)	.78 (>.99)	7.64 (2.53)	6.81 (2.31)	3.57 (36)	<.001 (.41)	7.69 (2.40)	6.81 (2.31)	3.88 (36)	<.001 (.34)
Least pain in past 24 h, mean (SD)	2.67 (2.03)	2.08 (1.49)	2.85 (38)	.007 (.35)	2.67 (2.03)	1.59 (1.33)	3.80 (38)	<.001 (.01)	2.08 (1.49)	1.59 (1.33)	1.98 (38)	<.001 (.58)	4.33 (2.25)	3.82 (1.60)	2.08 (38)	.04 (.71)	4.33 (2.25)	3.35 (1.80)	2.78 (36)	.009 (.08)	3.82 (1.60)	3.35 (1.80)	1.63 (36)	.11 (.86)
Pain right now, mean (SD)	4.21 (1.61)	3.85 (1.53)	2.16 (38)	.04 (.90)	4.21 (1.61)	2.82 (1.43)	5.72 (38)	<.001 (<.001)	3.85 (1.53)	2.82 (1.43)	3.72 (38)	<.001 (.01)	5.38 (1.87)	4.85 (1.87)	2.32 (38)	.03 (.51)	5.38 (1.87)	4.30 (1.37)	3.57 (36)	<.001 (.02)	4.85 (1.87)	4.30 (1.37)	1.96 (36)	.06 (.51)
WPI^c^ score, mean (SD)	17.54 (4.28)	12.59 (3.86)	11.51 (38)	<.001 (<.001)	17.54 (4.28)	7.28 (2.41)	21.24 (38)	<.001 (<.001)	12.59 (3.86)	7.28 (2.41)	12.16 (38)	<.001 (<.001)	16.46 (4.27)	13.56 (4.15)	9.72 (38)	<.001 (.01)	16.46 (4.27)	11.38 (3.96)	16.68 (36)	<.001 (<.001)	13.56 (4.15)	11.38 (3.96)	7.49 (36)	<.001 (.07)
SSS^d^, mean (SD)	6.54 (2.36)	4.95 (2.62)	5.75 (38)	<.001 (.005)	6.54 (2.36)	2.54 (1.39)	13.40 (38)	<.001 (<.001)	4.95 (2.62)	2.54 (1.39)	8.42 (38)	<.001 (<.001)	7.10 (2.49)	6.03 (3.19)	2.34 (38)	.02 (.24)	7.10 (2.49)	4.73 (2.29)	6.50 (36)	<.001 (<.001)	6.03 (3.19)	4.73 (2.29)	4.50 (36)	<.001 (.12)
FIQ^e^ score, mean (SD)	49.40 (12.71)	46.86 (11.55)	2.89 (38)	.006 (>.99)	49.40 (12.71)	44.68 (10.67)	3.74 (38)	<.001 (.23)	46.86 (11.55)	44.68 (10.67)	2.42 (38)	.02 (>.99)	50.98 (11.32)	49.93 (10.39)	1.25 (38)	.22 (>.99)	50.98 (11.32)	46.51 (10.76)	3.74 (36)	.01 (.22)	49.93 (10.39)	46.51 (10.76)	1.88 (36)	.07 (.52)
PSQI^f^ score, mean (SD)	18.67 (6.65)	17.85 (4.57)	<.001 (38)	.29 (>.99)	18.67 (6.65)	14.82 (5.43)	1.41 (38)	<.001 (.01)	17.85 (4.57)	14.82 (5.43)	5.08 (38)	<.001 (.06)	21.41 (5.92)	21.41 (6.29)	<.001 (38)	>.99 (>.99)	21.41 (5.92)	20.49 (5.52)	1.41 (36)	.17 (>.99)	21.41 (6.29)	20.49 (5.52)	2.71 (36)	.01 (>.99)

a T0: beginning of treatment; T1: 1 month after treatment; T3: 3 months after treatment.

b The value in parentheses is the adjusted *P* value (Bonferroni).

c WPI: Widespread Pain Index.

d SSS: symptom severity score.

e FIQ: Fibromyalgia Impact Questionnaire.

f PSQI: Pittsburgh Sleep Quality Index.

**Table 3.** Between-group differences of supervised versus unsupervised groups across time points (T0, T1, T3)^a^.

Evaluation metrics		T0	T1	T3
**Pain on average**				
	Supervised, mean (SD)	5.74 (2.51)	5.00 (2.28)	3.33 (1.75)
	Unsupervised, mean (SD)	6.28 (2.12)	5.87 (1.99)	5.14 (1.51)
	95% CI	−0.42 to 1.50	0.07 to 1.67	1.09 to 2.54
	*t* test (*df*)	1.14 (38)	2.21 (38)	5.07 (36)
	*P* value	.26	.03	<.001
**Worst pain in past 24 h**				
	Supervised, mean (SD)	7.08 (2.73)	6.62 (2.74)	5.36 (2.05)
	Unsupervised, mean (SD)	7.64 (2.53)	7.69 (2.40)	6.81 (2.31)
	95% CI	−0.44 to 1.57	0.14 to 2.02	0.63 to 2.24
	*t* test (*df*)	1.14 (38)	2.32 (38)	3.60 (36)
	*P* value	.26	.02	<.001
**Least pain in past 24 h**				
	Supervised, mean (SD)	2.67 (2.03)	2.08 (1.49)	1.59 (1.33)
	Unsupervised, mean (SD)	4.33 (2.25)	3.82 (1.60)	3.35 (1.80)
	95% CI	0.89 to 2.44	1.12 to 2.37	1.02 to 2.38
	*t* test (*df*)	4.36 (38)	5.68 (38)	5.08 (36)
	*P* value	<.001	<.001	<.001
**Pain right now**				
	Supervised, mean (SD)	4.21 (1.61)	3.85 (1.53)	2.82 (1.43)
	Unsupervised, mean (SD)	5.38 (1.87)	4.85 (1.87)	4.30 (1.37)
	95% CI	0.52 to 1.84	0.37 to 1.64	0.80 to 2.12
	*t* test (*df*)	3.64 (38)	3.19 (38)	4.48 (36)
	*P* value	<.001	<.001	<.001
**WPI^b^ score**				
	Supervised, mean (SD)	17.59 (4.40)	12.59 (3.86)	7.28 (2.41)
	Unsupervised, mean (SD)	16.46 (4.27)	13.56 (4.15)	11.38 (3.96)
	95% CI	−2.83 to 0.68	−0.50 to 2.45	2.73 to 5.49
	*t* test (*df*)	−1.24 (38)	1.34 (38)	6.03 (36)
	*P* value	.23	.19	<.001
**SSS^c^**				
	Supervised, mean (SD)	6.54 (2.36)	4.95 (2.62)	2.57 (1.43)
	Unsupervised, mean (SD)	7.10 (2.49)	6.03 (3.19)	4.73 (2.29)
	95% CI	−0.04 to 1.16	−0.10 to 2.26	1.39 to 2.93
	*t* test (*df*)	1.91 (38)	1.85 (38)	5.71 (36)
	*P* value	.06	.07	<.001
**FIQ^d^ score**				
	Supervised, mean (SD)	49.40 (12.71)	46.86 (11.55)	44.68 (10.67)
	Unsupervised, mean (SD)	50.98 (11.32)	49.93 (10.39)	46.51 (10.76)
	95% CI	−3.14 to 6.29	−1.45 to 7.57	−2.48 to 5.55
	*t* test (*df*)	0.68 (38)	1.38 (38)	0.78 (36)
	*P* value	.50	.18	.44
**PSQI^e^ score**				
	Supervised, mean (SD)	18.67 (6.65)	17.85 (4.57)	14.82 (5.43)
	Unsupervised, mean (SD)	21.41 (5.92)	21.41 (6.29)	20.49 (5.52)
	95% CI	−0.17 to 5.66	1.40 to 5.73	3.26 to 7.72
	*t* test (*df*)	1.9 (38)	3.33 (38)	4.99 (36)
	*P* value	.07	<.001	<.001

a T0: beginning of treatment; T1: 1 month after treatment; T3: 3 months after treatment.

b WPI: Widespread Pain Index.

c SSS: symptom severity score.

d FIQ: Fibromyalgia Impact Questionnaire.

e PSQI: Pittsburgh Sleep Quality Index.

**Figure 4.**
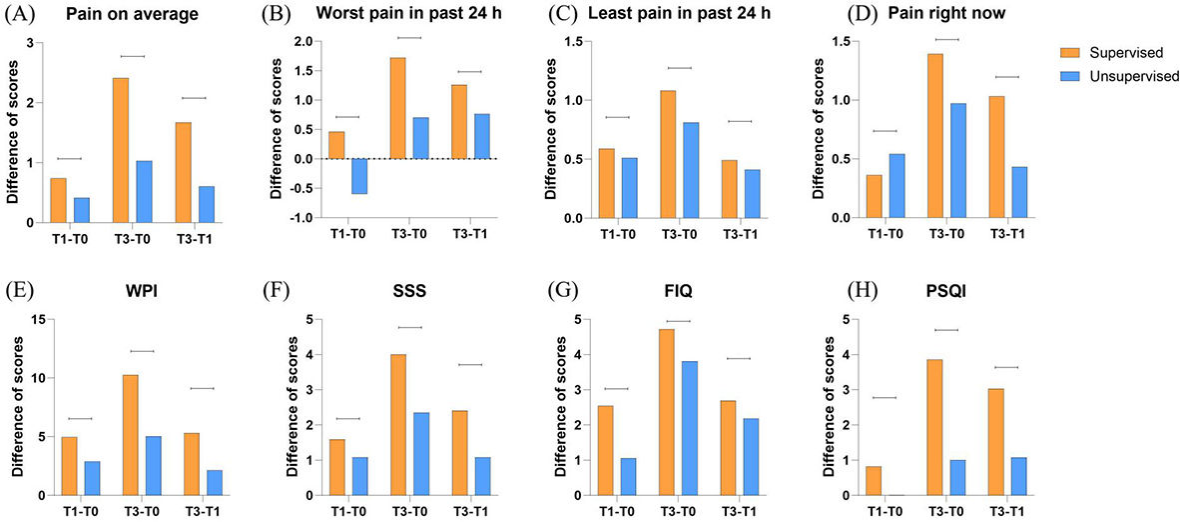
Difference in scores of BPI (pain on average, worst pain in past 24 h, least pain in past 24 h, pain right now), WPI, SSS, FIQ, and PSQI. BPI: Brief Pain Inventory; FIQ: Fibromyalgia Impact Questionnaire; PSQI: Pittsburgh Sleep Quality Index; SSS: symptom severity score; WPI: Widespread Pain Index. *P<.05. The specific *P* values are listed in Table 4.

However, the changes in scores from T0 to T1 (T1–T0), from T0 to T3 (T3–T0), and from T1 to T3 (T3–T1) in the supervised group were all less statistically significant compared to the unsupervised group (Table 4 and Figure 4).

**Table 4.** Between-group differences of supervised versus unsupervised groups across time intervals (T1–T0, T3–T0, and T3–T1)^a^.

Evaluation metrics		T1–T0	T3–T0	T3–T1
**Pain on average**				
	Supervised, mean (SD)	0.74 (1.21)	2.41 (1.43)	1.67 (1.08)
	Unsupervised, mean (SD)	0.41 (1.31)	1.03 (1.30)	0.60 (1.21)
	95% CI	−0.25 to 0.91	0.76 to 2.01	1.09 to 2.54
	*t* test (*df*)	1.14 (38)	2.21 (38)	5.07 (36)
	*P* value	.87	4.40	>.99
**Worst pain in past 24 h**				
	Supervised	0.46 (1.19)	1.72 (1.88)	1.26 (1.62)
	Unsupervised	−0.51 (1.15)	0.70 (1.20)	0.76 (1.19)
	95% CI	0.44 to 1.51	0.30 to 1.74	−0.16 to 1.16
	*t* test (*df*)	3.61 (38)	2.82 (38)	1.51 (36)
	*P* value	>.99	>.99	.93
**Least pain in past 24 h**				
	Supervised	0.59 (1.29)	1.08 (1.77)	0.49 (1.54)
	Unsupervised	0.51 (1.54)	0.81 (1.78)	0.41 (1.52)
	95% CI	−0.57 to 0.73	−0.54 to 1.08	−0.63 to 0.79
	*t* test (*df*)	0.25 (38)	0.66 (38)	0.23 (36)
	*P* value	.60	.75	.59
**Pain right now**				
	Supervised	0.36 (1.04)	1.39 (1.58)	1.03 (1.72)
	Unsupervised	0.54 (1.45)	0.97 (1.66)	0.43 (1.35)
	95% CI	−0.76 to 0.40	−0.32 to 1.16	−0.12 to 1.32
	*t* test (*df*)	−0.62 (38)	1.13 (38)	1.66 (36)
	*P* value	.27	.87	.95
**WPI^b^ score**				
	Supervised	4.95 (2.69)	10.25 (3.02)	5.31 (2.73)
	Unsupervised	2.90 (1.86)	5.03 (1.83)	2.14 (1.74)
	95% CI	0.99 to 3.11	4.08 to 6.36	2.10 to 4.24
	*t* test (*df*)	3.86 (38)	9.11 (38)	5.92 (36)
	*P* value	>.99	>.99	>.99
**SSS^c^**				
	Supervised	1.59 (1.73)	4.00 (1.86)	2.41 (1.79)
	Unsupervised	1.08 (2.87)	2.35 (2.20)	1.08 (1.46)
	95% CI	−0.57 to 1.59	0.72 to 2.58	0.57 to 2.09
	*t* test (*df*)	0.94 (38)	3.53 (38)	3.49 (36)
	*P* value	.82	>.99	>.99
**FIQ^d^ score**				
	Supervised	2.54 (5.49)	4.72 (7.89)	2.69 (8.69)
	Unsupervised	1.05 (5.26)	3.81 (8.46)	2.18 (5.64)
	95% CI	−0.97 to 3.95	−2.83 to 4.65	−2.91 to 3.93
	*t* test (*df*)	1.21 (38)	0.49 (38)	0.30 (36)
	*P* value	.89	.69	.62
**PSQI^e^ score**				
	Supervised	0.82 (4.74)	3.85 (5.30)	3.03 (3.72)
	Unsupervised	0.00 (4.45)	1.00 (4.33)	1.08 (2.43)
	95% CI	−1.28 to 2.92	0.64 to 5.06	0.49 to 3.42
	*t* test (*df*)	0.78 (38)	2.57 (38)	2.66 (36)
	*P* value	.78	.99	>.99

a T0: beginning of treatment; T1: 1 month after treatment; T3: 3 months after treatment.

b WPI: Widespread Pain Index.

c SSS: symptom severity score.

d FIQ: Fibromyalgia Impact Questionnaire.

e PSQI: Pittsburgh Sleep Quality Index.

### Safety Assessment

Both groups of patients with fibromyalgia did not experience adverse reactions related to drug treatment, such as dizziness, nausea, and drowsiness, nor did they experience adverse reactions to exercise treatment, such as headaches, blurred vision, chest tightness, and sports injuries.

## Discussion

### Principal Findings

In this study, we found that compared with before treatment, the overall pain condition (BPI score, WPI score, SSS), quality of life (FIQ score), and sleep condition (PSQI score) of the 2 groups were significantly changed, indicating that exercise combined with medication led to favorable improvements in somatic symptoms, psychiatric symptoms, quality of life, pain, and sleep disturbances in patients treated for FM. The scores of the 2 groups of patients were further reduced at 3 months after treatment compared with 1 month after treatment. It can be seen that when exercise is combined with drug treatment, as the treatment time grows longer, the effect becomes noticeable and has good synergy. However, after applying Bonferroni correction for multiple comparisons across time points, some of the previously significant differences between the 2 groups were no longer statistically significant (*P*≥.05). This is likely due to the conservative nature of the Bonferroni correction, which adjusts the significance threshold to reduce the risk of type I errors. The loss of significance at certain time points suggests that some initial findings may have been false positives, and the remaining significant results after correction should be prioritized in the interpretation of the study outcomes. Future studies could consider alternative correction methods, such as the Holm-Bonferroni approach or false discovery rate control, to better balance the trade-off between type I and type II errors. Additionally, a larger sample size in future studies may improve the statistical power to detect true differences. The changes in scores from T0 to T1 (T1–T0) in the supervised group were all less statistically significant compared to the unsupervised group, indicating that observation over a longer period of time and of a larger sample is needed. The same changes were seen at T3.

In addition, 3 months after treatment, 2 patients in the unsupervised group withdrew from the trial due to their inability to persist with the exercise program, while all patients in the supervised group were able to persist in the exercises. We speculate that it is precisely because the unsupervised group did not use a remote network application to supervise their exercise and the patient’s compliance was poor that the unsupervised group found it more difficult to persist than the supervised group, further confirming the necessity of supervised exercise. At the same time, neither group of patients had adverse reactions related to either the drug or exercise treatment. Previous studies have also not reported adverse reactions to drug therapy [5]. It does not indicate that the incidence of adverse reactions to exercise combined with drug therapy is lower than that of drug therapy alone. This needs to be further confirmed by studies with larger sample sizes. Therefore, remote supervised exercise therapy combined with medication is safe, effective, and easy to implement for patients with fibromyalgia.

Our findings are consistent with previous studies that have highlighted the benefits of exercise therapy in managing FM symptoms. For instance, a meta-analysis demonstrated that supervised exercise programs significantly reduce pain and improve quality of life in patients with fibromyalgia [15]. However, our study extends these findings by incorporating remote network supervision, which enhances adherence and allows for real-time adjustments, a feature not extensively explored in prior research. Unlike traditional in-person supervision, our approach leverages technology to provide continuous support, potentially addressing the high dropout rates often observed in long-term exercise interventions for patients with fibromyalgia.

The observed improvements in pain, sleep quality, and mental health observed in our study are also associated with several mechanisms attributed to other studies. First, regular exercise is known to modulate central pain processing by reducing central sensitization, a key pathological feature of FM [19-21]. Second, exercise-induced release of endorphins and other neurochemicals may contribute to mood enhancement and pain relief [22]. Third, the structured nature of the supervised program, combined with real-time feedback, likely improved patient engagement and self-efficacy, which are critical for long-term symptom management [23]. These mechanisms align with the biopsychosocial model of FM, which emphasizes the interplay between physical, psychological, and social factors in symptom progression and management.

The study still has some limitations. This study is a single-center study with relatively few patient samples. Although it was found that exercise combined with drug therapy can significantly improve the clinical symptoms of FM, and long-term supervised exercise training is more effective than unsupervised, further multicenter, large-sample studies are needed to confirm this to provide stronger evidence for FM treatment guidelines. Second, our study was single-blinded, but neither the researchers nor the data analysts were fully blinded, which may have introduced potential bias. This limitation may have affected the objectivity of the data collection and analysis. We chose a single-blind design due to practical constraints, such as the difficulty of blinding the researchers to the exercise intervention, as the intervention itself was visible and required the active participation of both patients and researchers. In future studies, we will consider a double-blind design to minimize bias. For example, exercise interventions will be delivered by independent rehabilitation therapists not affiliated with the research team, who will only know group codes. Researchers involved in data collection and analysis could be blinded to group assignment, while independent researchers could oversee the implementation of the exercise intervention, using standardized operating procedures and unified training for all assessors. Anonymized raw data could be transferred to an independent statistical team, with group labels withheld (referred to as Group A/B) prior to analysis. Objective indicators would be prioritized, while subjective measures (eg, pain) would undergo third-party blinded assessments, such as remote review of patient diaries by external experts. These steps aim to minimize bias.

Regarding the group design of our study, this research focused on exploring the differences between “supervised” and “unsupervised” exercise modalities. The absence of a medication-only control group represents a limitation, which we determined based on 2 considerations: first, FM treatment guidelines emphasize the necessity of combined exercise interventions; second, establishing a medication-only group appeared ethically questionable given the established benefits of exercise interventions for FM. In future studies, we plan to conduct subgroup analyses of patients with varying medication usage intensities to further investigate exercise intervention efficacy. Although the advantages of this remote network supervision system are undeniable, there are also some challenges, such as ensuring that patients of different ages and educational levels can use the application for exercise training without obstacles, as well as ensuring patient data security and privacy protection. Therefore, the system needs to be continuously optimized and improved to adapt to the ever-changing medical environment and patient needs. In this study, WPI, SSS, BPI, FIQ, and PSQI scores were introduced with the aim of exploring the application of these scales in the diagnosis and observation of FM. However, it was found that since some scales were designed for psychiatry, focusing on psychiatric symptomatic factors, bias was inevitable in their use for FM, especially for PSQI scales, which could not be fully reflected in the scale scores, even though the symptoms of patients with FM had been significantly improved in the clinic. This is related to the lack of somatic design factors in the PSQI scale. However, from the results of this study on sleep improvement, the PSQI scale score has a high reliability.

No adverse events related to pharmacological treatment or exercise therapy were observed in this study, which may partially reflect the safety of the interventions, but could also be associated with other factors: (1) Adverse event reporting in this study primarily relied on patient self-reports during follow-up visits. Standardized adverse event scales or dynamic physiological monitoring were not systematically implemented, potentially leading to underdetection of mild/subclinical reactions. (2) The exercise intervention protocol strictly adhered to FM exercise guidelines, using progressive low-intensity aerobic training, thereby inherently reducing risks associated with high-intensity exercise [24]. (3) All medications (eg, pregabalin) were approved agents, with dose adjustments based on individual tolerance to minimize acute adverse reactions. (4) Strict exclusion criteria (eg, exclusion of high-risk populations with severe cardiopulmonary diseases or hepatic/renal dysfunction) resulted in a study cohort with lower baseline risk, limiting the occurrence of adverse events. (5) The 3-month study duration and 4-week follow-up intervals may have precluded detection of long-term adverse effects or rare incidents. In future studies, extending the follow-up period and integrating active monitoring tools (eg, wearable devices with automated detection of signals such as abrupt impacts) could further enhance safety assessments.

### Conclusion

In conclusion, the program of exercise combined with drug therapy for FM under the supervision and guidance of a remote network application not only showed significant positive effects in pain relief, sleep quality improvement, improvement of somatic and mental symptoms, and improvement of quality of life, but also showed more obvious and safe improvement of FM symptoms through exercise training under long-term supervision. This treatment program is forward-looking and innovative. It is tailored for patients with fibromyalgia and offers personalized exercise plans. In addition, it enables real-time supervision and dynamic adjustment to minimize adverse events. This study underscores the potential of integrating technology into FM management, offering a scalable and accessible solution for patients who may face barriers to in-person care. The personalized and supervised approach not only enhances treatment efficacy but also empowers patients to take an active role in their recovery. As remote health care continues to evolve, our findings highlight the importance of innovative, patient-centered interventions in chronic disease management.

## Supplementary material

10.2196/71624 Checklist 1 CONSORT-EHEALTH checklist (V 1.6.1).

## Abbreviations

BPI: Brief Pain Inventory
FIQ: Fibromyalgia Impact Questionnaire
FM: fibromyalgia
PSQI: Pittsburgh Sleep Quality Index
SSS: symptom severity score
T0: beginning of treatment
T1: 1 month after treatment
T3: 3 months after treatment
WPI: Widespread Pain Index
